# Transjugular intrahepatic portosystemic shunt for hepatic sinusoidal obstruction syndrome with primary biliary cholangitis and alcoholic liver disease: a case report

**DOI:** 10.3389/fmed.2025.1696892

**Published:** 2025-11-27

**Authors:** Pei Yang, Feng Zhang, Feng Wang, Yang He, Yao Wang, Lingrong Qian

**Affiliations:** 1 Department of Digestive Endoscopy, Affiliated Qujing Second People's Hospital, Qujing, Yunnan, China; 2 Department of Gastroenterology, Affiliated Drum Tower Hospital of Nanjing University Medical School, Nanjing, Jiangsu, China; 3 Department of Clinical Nutrition, Affiliated Qujing Second People's Hospital, Qujing, Yunnan, China

**Keywords:** hepatic veno-occlusive disease/sinusoidal obstruction syndrome, primary biliary cholangitis, alcohol-associated liver disease, transjugular intrahepatic portosystemic shunt, pyrrolizidine alkaloids, multi-etiology liver injury

## Abstract

This article reports the case of a 73-year-old man with a 50-year history of long-term alcohol abuse (with daily intake of 250 g) combined with primary biliary cholangitis (PBC, AMA-M2 positive). The patient recently developed refractory ascites and manifestations of portal hypertension (hepatic venous pressure gradient, HVPG: 17.3 mmHg). Liver biopsy revealed characteristic sinusoidal dilation and congestion, fibrosis, and erythrocyte extravasation. It was discovered that the patient had a history of consuming *Gynura segetum* (a traditional Chinese herb) for 1 year. Based on clinical presentation and diagnostic findings, the final diagnosis included the following: (1) hepatic veno-occlusive disease/sinusoidal obstruction syndrome (VOD/SOS), (2) portal hypertension, (3) primary biliary cholangitis (PBC), and (4) alcoholic cirrhosis. After a poor response to medical treatment, a transjugular intrahepatic portosystemic shunt (TIPS) was performed, resulting in significant improvement (postoperative portal pressure gradient (PPG) decreased to 8 mmHg, with complete resolution of ascites). During a 6-month follow-up, liver function showed marked improvement, with the Child–Pugh score improving from class C (11 points) to class A (6 points).

## Introduction

Hepatic sinusoidal obstruction syndrome (SOS), also known as hepatic veno-occlusive disease (VOD), is a severe hepatic disorder pathologically characterized by fibrous obliteration of small hepatic veins, endothelial damage to hepatic sinusoids, and parenchymal congestion—resulting in acute portal hypertension. Clinically, it manifests as right upper quadrant pain, hepatomegaly, ascites, and jaundice (1). This condition has a complex pathogenesis with diverse etiologies, primarily including hematopoietic stem cell transplantation-related complications, cytotoxic agents (e.g., oxaliplatin), and intoxication from pyrrolizidine alkaloid (PA)-containing herbal medicines (such as *Gynura segetum*), which is particularly common in China (2, 3).

Pyrrolizidine alkaloid-induced hepatic sinusoidal obstruction syndrome (PA-HSOS) can be divided into acute, subacute, and chronic stages. The acute type is characterized by the rapid onset of hepatomegaly, painful ascites, and jaundice; the subacute type presents with persistent hepatomegaly and ascites over several weeks, while the chronic type leads to portal hypertension and can progress to cirrhosis (4). Early supportive treatments, such as anticoagulation therapy, liver protection (e.g., with pharmaceutical preparations including ursodeoxycholic acid and polyene phosphatidylcholine), jaundice reduction, albumin and plasma supplementation, diuresis, and ascites control, can lead to improvement in over 50% of patients with acute or subacute stages (5). In contrast, patients in the chronic phase often require lifelong management of portal hypertension complications, such as refractory ascites and variceal bleeding.

Previous studies have shown that, for patients who do not respond to anticoagulant therapy, the overall effective rate of subsequent treatment with transjugular intrahepatic portosystemic shunt (TIPS) is as high as 91%. TIPS can significantly improve the quality of life and survival of critically ill patients (5). However, the therapeutic strategy and long-term outcomes of TIPS in patients with complex liver injury remain insufficiently investigated. In this case report, we describe for the first time the successful treatment of refractory ascites—caused by the combined etiologies of PA-HSOS, primary biliary cholangitis (PBC), and alcohol-associated liver disease (ALD)—using TIPS.

## Case report

**Figure 1:**
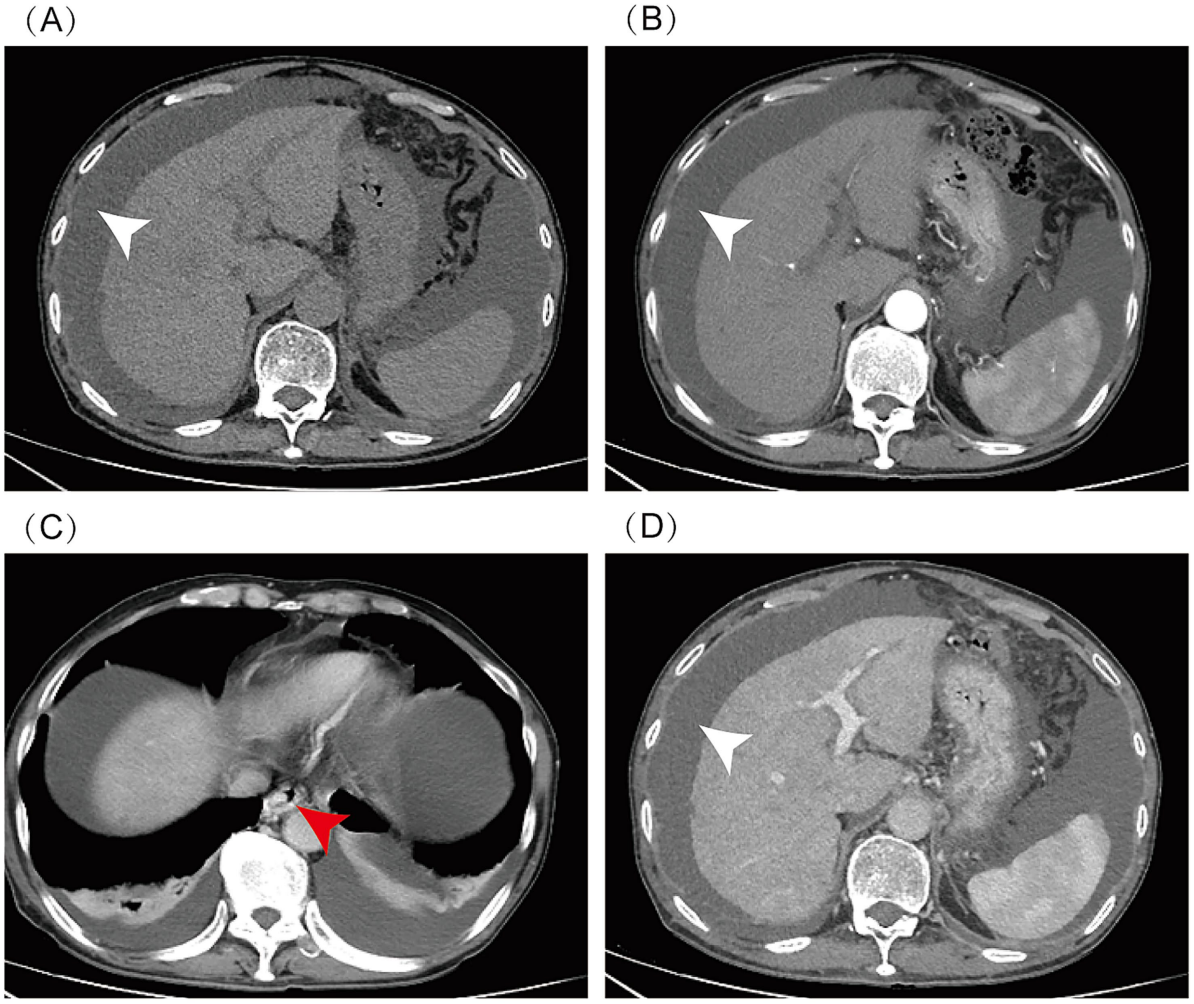
Abdominal enhanced CT: massive ascites (indicated by white arrow), cirrhosis, esophageal and gastric varices (indicated by red arrow), and hepatic steatosis. CT scan phases: **(A)** Non-contrast, **(B)** Arterial, **(C)** Venous, **(D)** Delayed.

In November 2024, a 73-year-old man was admitted to our hospital with the chief complaint of “abdominal distension for over half a month.” The patient had a 50-year history of chronic alcohol abuse (daily alcohol intake of 250 g), primary biliary cholangitis (PBC, AMA-M2 positive), and a 1-year history of consuming *Gynura segetum*-infused alcohol. Laboratory examinations showed alanine aminotransferase (ALT) of 14.7 U/L, aspartate aminotransferase (AST) of 54.5 U/L, alkaline phosphatase (ALP) of 286.4 U/L, *γ*-glutamyl transferase (GGT) of 697.7 U/L, lactate dehydrogenase (LDH) of 305 U/L, total bilirubin of 80.3 μmol/L, direct bilirubin of 29.7 μmol/L, albumin of 38.2 g/L, blood ammonia of 35.9 μmol/L; prothrombin time of 13.8 s, international normalized ratio (INR) of 1.21; and anti-AMA-M2 antibody positive (+). Enhanced abdominal computed tomography (CT) revealed cirrhosis, massive ascites, dilated portal vein, esophageal and gastric varices, and hepatic steatosis (Figure 1). Upper gastroscopy showed grade II–III esophageal varices and portal hypertensive gastropathy. Ascites examination: ascitic fluid somatic cell count (AF-SCC) 340 × 10^6^/L, total protein 21.3 g/L, albumin 13.8 g/L, and adenosine deaminase 4.6 U/L. The patient was managed with complete alcohol abstinence and received a combination of diuretic therapy, ursodeoxycholic acid, and carvedilol. Following symptomatic treatment with clinical improvement in abdominal distension, the patient was discharged.

**Figure 2:**
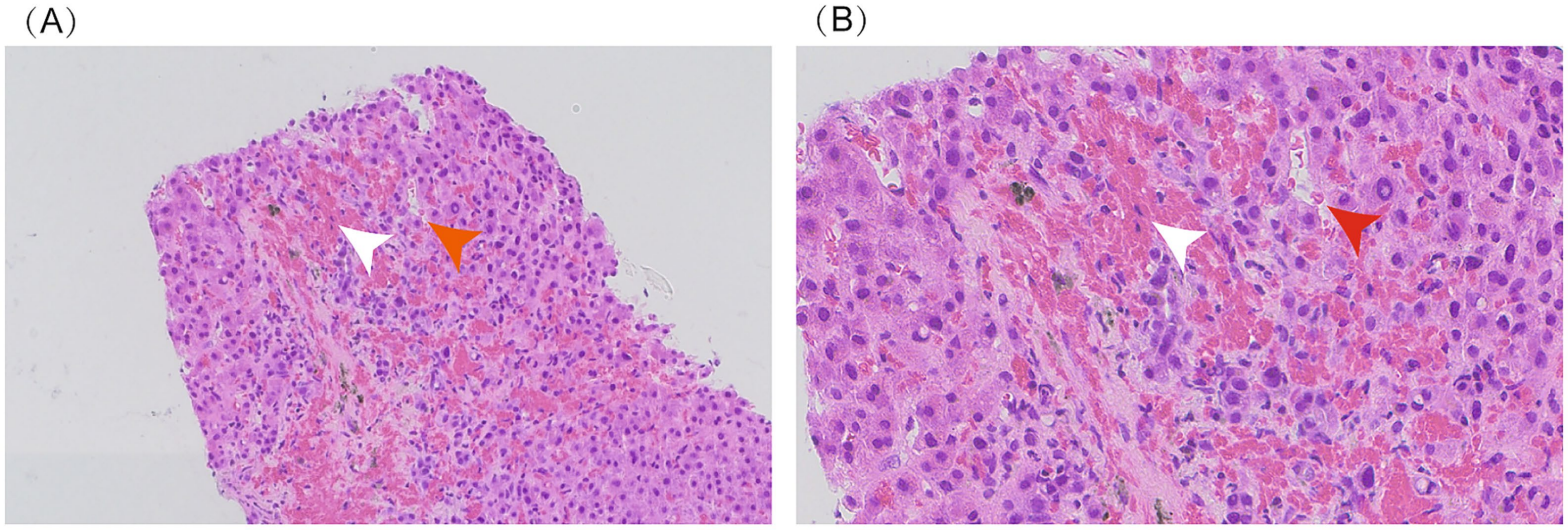
Liver biopsy: sinusoidal dilation and congestion (indicated by red arrow) and fibrous tissue hyperplasia with red blood cell extravasation (indicated by white arrow). Panel **A** (20x magnification); Panel **B** (40x magnification).

Approximately 1 month later, the patient suffered recurrent and progressively worsening abdominal distension and massive ascites and was readmitted. Hepatic venous pressure gradient (HVPG) measurement and transjugular liver biopsy (TJLB) were performed to confirm the diagnosis. The HVPG was 17.3 mmHg, which was significantly elevated. The pathology from TJLB showed sinusoidal dilatation and congestion, mild hepatocellular edema, chronic inflammatory cell infiltration, focal lymphocytic infiltration, mild interface hepatitis, and degeneration and loss of small interlobular bile ducts (Figure 2), indicating a complex etiology involving drug-induced liver injury and PBC combined with alcohol-related liver injury. Based on these data, the patient was definitively diagnosed with SOS complicated by PBC and ALD. Despite therapeutic interventions, including hepatoprotective agents, a triple diuretic regimen, and paracentesis, the patient’s abdominal distension showed minimal improvement, meeting criteria for refractory ascites.

**Figure 3:**
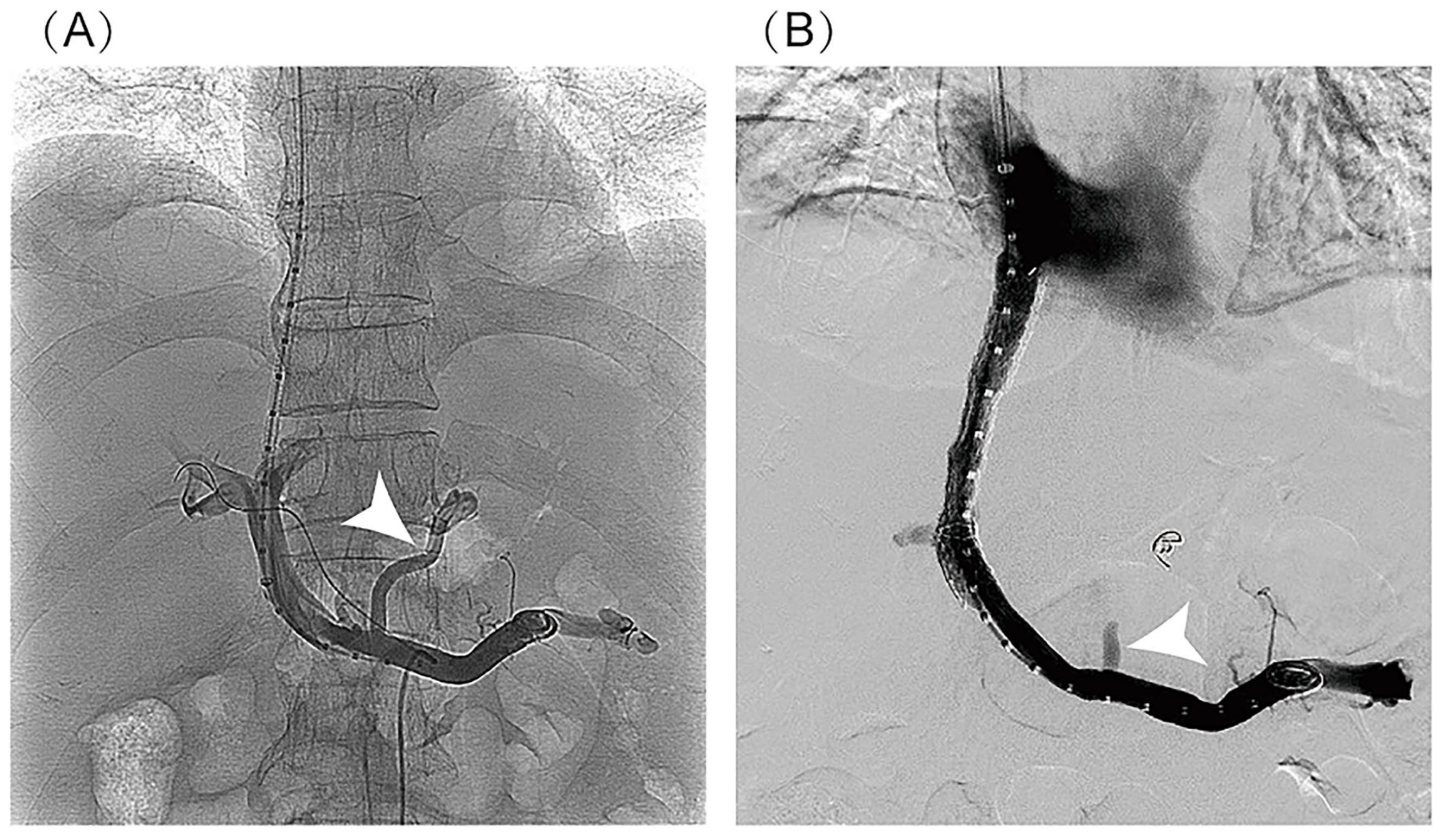
Imaging findings of TIPS procedure: **(A)** post-puncture portal venography: It clearly demonstrates the normal anatomical course of the main portal vein and its intrahepatic branches, with homogeneous and complete contrast opacification of the portal venous system. No significant luminal stenosis, thrombosis, or contrast extravasation is observed. Portal venous collaterals are visualized (indicated by the white arrow). **(B)** Post-stent venography: It shows patient blood flow through the stent with a well-defined portal-to-hepatic venous shunt pathway. No contrast stasis or extravasation is noted. Previously visualized collateral vessels have disappeared (indicated by the white arrow). The stent demonstrates optimal morphology, appropriate positioning, and complete expansion.

In March 2025, the patient underwent TIPS for refractory ascites with portal hypertension unresponsive to medical therapy (Figure 3). Preoperative portal pressure gradient (PPG) was 23 mmHg. A Viatorr-controlled expandable stent (8–10 mm × 70 + 20 mm) was successfully placed and dilated with a 6-mm balloon in size. Immediate PPG decreased to 7 mmHg. After TIPS, the patient recovered quickly from massive ascites, with stable liver function (total bilirubin: 96 μmol/L). During the 6-month post-procedural follow-up, the patient reported resolution of abdominal distension and recovery of walking ability, with a satisfaction score of 4/5 and significant improvement in quality of life. Additionally, the patient’s liver function showed gradual recovery (total bilirubin: 60.8 μmol/L; albumin 39.2 g/L; INR: 1.28), with the Child–Pugh score improving from class C (11 points) to class A (6 points). The TIPS stent remained patent, and no ascites or overt hepatic encephalopathy was observed (Table 1).

**Table 1:** Changes in LFTs and child–pugh score in a patient pre- and post-TIPS.

Time point	ALT (U/L)	AST (U/L)	TBIL (μmol/L)	ALB (g/L)	Child–pugh score (points)
10 months after *Gynura segetum* ingestion (December 2024)	19.4	54.5	80.3	38.2	8
11 months after *Gynura segetum* ingestion (January 2025)	13.6	32.1	87.7	34.5	10
12 months after *Gynura segetum* ingestion (February 2025)	24.6	42.0	98.8	33.1	10
13 months after *Gynura segetum* ingestion (Pre-TIPS) (March 2025)	14.4	25.5	105.5	27.6	11
1-month post-TIPS	18.8	48.4	77.1	35.6	8
3-month post-TIPS	19.9	46.3	62.9	38.1	6
6-month post-TIPS	11.5	26.0	60.8	39.2	6

1. Reference Range Explanation: The normal range of alanine aminotransferase (ALT) is 9–50 U/L, aspartate aminotransferase (AST) is 15–40 U/L, total bilirubin (TBIL) is ≤26 μmol/L, and albumin (ALB) is 40–55 g/L.

2. Child–Pugh Score Explanation: This score is used to assess liver function reserve in patients with cirrhosis, calculated based on five indicators (total bilirubin, albumin, prothrombin time, ascites, and hepatic encephalopathy). It ranges from 5 to 15 points, with higher scores indicating more severe liver dysfunction (5–6 points: grade A; 7–9 points: grade B; 10–15 points: grade C).

3. Data Source: All indicator data are derived from the hospital’s electronic medical record system to ensure authenticity and traceability.

4. Note on Gynura segetum Ingestion Time: The time of Gynura segetum ingestion was confirmed by the patient’s recall, accurate for the specific month.

## Discussion

Drug-induced liver injury (DILI) is frequently caused by herbal medicines and dietary supplements. Numerous pyrrolizidine alkaloid (PA)-containing herbs can induce liver injury, including *Senecio scandens*, *Senecio vulgaris*, and *Gynura segetum* (3). PA-HSOS presents with an acute onset, severe condition and a high fatality rate, with a lack of effective treatment options. However, if diagnosis and treatment are delayed, 20% of patients may die from acute liver failure (4). Patients with mild-to-moderate PA-HSOS are more likely to benefit from anticoagulation therapy (6). However, studies report non-response rates of 30.17% in mild cases and 33.82% in moderate cases (7, 8). For anticoagulation-refractory patients, subsequent TIPS achieved an overall response rate of 91%, especially for those with a higher level of total bilirubin. Consequently, the currently recommended therapeutic strategy for PA-HSOS follows an “anticoagulation-TIPS stepwise approach” (5).

This study reports for the first time a complex liver injury case caused by three combined etiologies: SOS, PBC, and ALD. The patient’s imaging examination did not show the characteristic CT features of SOS, which once made the initial diagnosis difficult. Due to its non-specific presentation and relative rarity, PA-HSOS is often misdiagnosed (9). In this patient, the final diagnosis was confirmed through liver biopsy (showing sinusoidal vascular congestion) and direct hemodynamic measurement via hepatic venous pressure gradient (HVPG), in accordance with the 2017 “Nanjing Criteria” (10).

For patients with refractory ascites caused by simple ALD or PBC who have good liver function, TIPS has a significant effect on improving complications and survival (11). However, if liver function is poor, especially with a markedly elevated total bilirubin, the patients often cannot benefit from TIPS. In the present case, the patient had severely impaired liver function; however, the distinct contributions of the three etiologies could not be validated. If the patient had only ALD or PBC, the risk of liver failure caused by TIPS would be extremely high. However, this case was complicated by PA-HSOS. Hyperbilirubinemia and jaundice are almost invariably present in classic SOS/VOD in adult patients (12). Previous studies have shown that portal hypertension in ALD/PBC is primarily caused by liver fibrosis and the formation of pseudolobules (13). Although TIPS can reduce portal pressure, it cannot reverse hepatocellular necrosis or liver failure. In contrast, the hepatic sinusoidal obstruction in SOS is reversible, and TIPS can promote hepatocellular repair by improving hemodynamics. TIPS is a safe and effective therapeutic strategy for PA-HSOS patients who do not respond to conservative treatment (14). In this patient, the markedly elevated HVPG (17.3 mmHg) suggested portal hypertension as the primary pathophysiological mechanism, indicating that the benefits of TIPS may outweigh the risks. Although liver transplantation is a curative option, the patient’s ongoing alcohol use is a relative contraindication. A multidisciplinary approach to evaluating TIPS candidacy is recommended to optimize outcomes (15). After a comprehensive assessment by a multidisciplinary team (hepatologists, interventional radiologists, and transplant surgeons), TIPS was determined as the treatment. The team also hypothesized that TIPS could effectively alleviate severe portal hypertension—primarily caused by potentially reversible hepatic sinusoidal obstruction syndrome (SOS)—thus facilitating liver function restoration. After TIPS, the PPG decreased significantly from 21 mmHg to 8 mmHg, the ascites symptoms improved significantly, and liver function continued to recover during the 6-month follow-up. The therapeutic mechanism likely involves effective portal-systemic shunting that reduces sinusoidal pressure and improves hepatic microcirculatory dysfunction.

It is necessary to recognize the limitations of this single-case report. Individual differences cannot be ruled out as factors influencing the treatment outcome, and it is difficult to verify the universal applicability of TIPS in similar patients. This report also highlights that conventional liver function (e.g., Child–Pugh and Model for End-Stage Liver Disease (MELD)) scores may have limitations in assessing TIPS risks when multiple, mechanistically distinct liver diseases coexist. A comprehensive preoperative evaluation—encompassing liver function, precise disease etiology, and hemodynamic status—is essential to formulate individualized treatment strategies that maximize benefits and mitigate risks.

## Data Availability

The original contributions presented in the study are included in the article/supplementary material, further inquiries can be directed to the corresponding author/s.
